# Low-Frequency Noise Characteristics in HfO_2_-Based Metal-Ferroelectric-Metal Capacitors

**DOI:** 10.3390/ma15217475

**Published:** 2022-10-25

**Authors:** Ki-Sik Im, Seungheon Shin, Chan-Hee Jang, Ho-Young Cha

**Affiliations:** 1 Department of Green Semiconductor System, Daegu Campus, Korea Polytechnics, Daegu 41765, Korea; 2 School of Electronic and Electrical Engineering, Hongik University, Seoul 04066, Korea

**Keywords:** low-frequency noise, HfO_2_, ferroelectric

## Abstract

The transport mechanism of HfO_2_-based metal-ferroelectric-metal (MFM) capacitors was investigated using low-frequency noise (LFN) measurements for the first time. The current–voltage measurement results revealed that the leakage behavior of the fabricated MFM capacitor was caused by the trap-related Poole–Frenkel transport mechanism, which was confirmed by the LFN measurements. The current noise power spectral densities (*S_I_*) obtained from the LFN measurements followed 1/*f* noise shapes and exhibited a constant electric field (*E*) × *S_I_*/*I*^2^ noise behavior. No polarization dependency was observed in the transport characteristics of the MFM capacitor owing to its structural symmetry.

## 1. Introduction

Hafnium oxide (HfO_2_)-based ferroelectric (FE) materials can be used in memory devices and negative capacitance field-effect transistors (FETs) [1,2,3,4]. Owing to their outstanding material properties, such as a large dielectric constant of 20–25 and a high-energy bandgap of ~5.7 eV, HfO_2_-based metal-ferroelectric-metal (MFM) capacitors have high capacitance, low leakage current, and strong ferroelectric properties. HfO_2_ ferroelectric films can be deposited using an atomic layer deposition (ALD) system in a complementary metal-oxide-semiconductor (CMOS)-compatible process with excellent thickness controllability and large-area uniformity. 

Current–voltage (I–V) and capacitance–voltage (C–V) measurements are commonly used to evaluate MFM capacitors. The ferroelectric properties of HfO_2_-based MFM capacitors based on I–V and C–V measurements have also been reported as functions of frequency and temperature [5]. Giusi et al. [6] reported Ru-based capacitors characterized by low-frequency noise (LFN) measurements. LFN measurements can be used to analyze the conduction mechanism and evaluate the device/material reliability [7,8,9]. Recently, Shin et al. reported a study on a metal-FE-insulator-semiconductor (MFIS) FET based on noise measurements [7,8,9]. They investigated conduction mechanisms and noise fluctuation depending on the FE polarization direction and the process conditions. The MFIS configuration is asymmetric, with an additional interface between the FE and the semiconductor, whereas the MFM device has a symmetric interface between the FE and the metal electrodes. FE polarization has been reported to play a significant role in MFIS FET noise behavior; hence, analyzing FE material is important. However, LFN characteristics have not been reported for HfO_2_-based MFM capacitors. This study comprehensively analyzed a HfO_2_-based MFM capacitor using LFN measurements.

## 2. Device Fabrication

**Figure 1:**
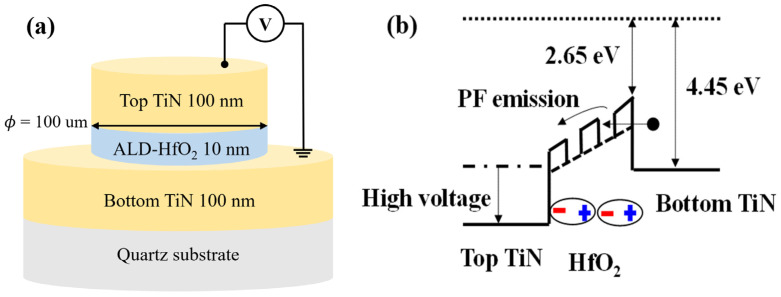
(**a**) Schematic of the fabricated HfO_2_-based MFM capacitor. (**b**) Illustration of the transport mechanism in the MFM device with an applied voltage.

**Figure 2:**
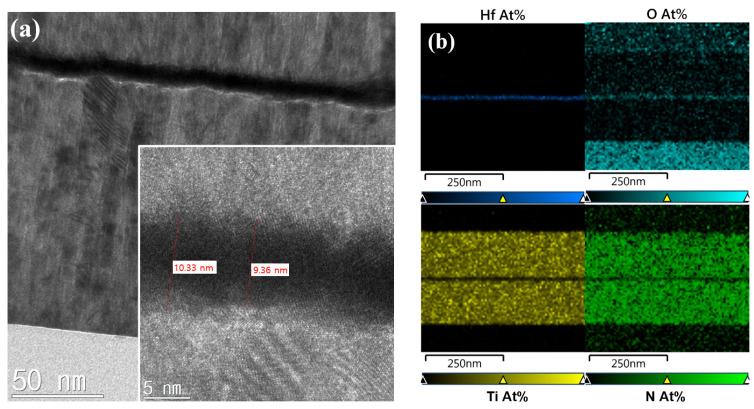
(**a**) Cross-sectional TEM images. (**b**) EDS analysis of the fabricated HfO_2_-based MFM capacitor.

The MFM capacitor consisted of 100-nm-thick TiN metal, 10-nm-thick HfO_2_ ferroelectric oxide, and 100-nm-thick TiN metal layers grown on a quartz substrate (Figure 1a). The fabrication process was as follows: After cleaning the wafer in wet chemical solutions (acetone, methanol, and deionized water), a 100-nm-thick TiN film was deposited at room temperature using a radio frequency (RF) sputtering machine. The bottom electrode pattern was defined using a fluorine-based plasma-etching process. A 10-nm-thick un-doped HfO_2_ ferroelectric layer was deposited via an atomic layer deposition (ALD) method at 220 °C using a TEMA-Hf precursor and 100 g/m^3^ of O_3_. Then, a 100-nm-thick TiN film was deposited as the top electrode, which was patterned with a diameter of 100 μm. The top TiN film and the un-doped HfO_2_ were subsequently etched to form capacitors using SF_6_/Ar and CF_4_/O_2_ gas mixtures. The fabricated MFM capacitors were annealed using rapid thermal annealing (RTA) at 650 °C for 1 min to obtain the desired ferroelectric properties. Cross-sectional transmission electron microscopy (TEM) images and energy-disperse spectroscopy (EDS) analysis of the fabricated MFM capacitor (shown in Figure 2) confirm the un-doped HfO_2_ of a thickness of 10 nm. Detailed ferroelectric characteristics, including X-ray diffraction analysis, can be found in ref. [5].

## 3. Characterization and Discussions

The I–V and LFN characteristics were measured in a shielding box at atmospheric pressure using a NOISYS7 machine (Synergie-concept) [10]. We measured fifteen devices from three samples (five from each sample), and no significant variation was observed. 

**Figure 3:**
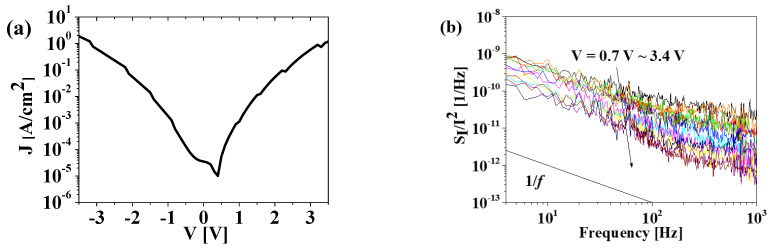
(**a**) Logarithmic scale of current density as a function of voltage in the MFM capacitor. (**b**) Normalized current power spectral density (*S_I_*/*I*^2^) versus frequency as a function of bias voltage ranging from 0.7 to 3.4 V.

**Figure 4:**
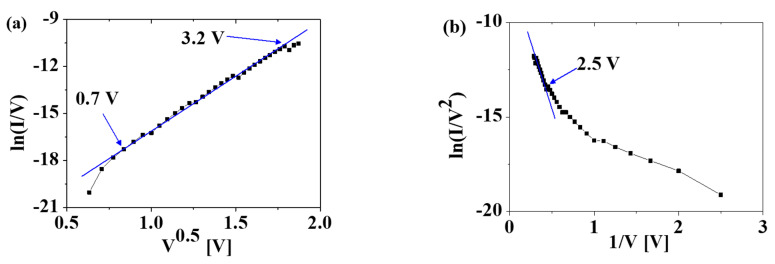
(**a**) ln (I/V) versus V^0.5^ and (**b**) ln (I/V^2^) versus 1/V show the Poole–Frenkel emission and the Fowler–Nordheim emission, respectively. From the straight lines, the dominant leakage mechanisms are the Poole–Frenkel mechanism from 0.7 to 3.2 V and the Fowler–Nordheim mechanism at V > 2.5 V.

Figure 3a shows the leakage current density (*J*) as a function of the bias voltage of the fabricated MFM capacitor. The leakage current behavior in both the forward and reverse-bias regimes exhibits similar rectifying characteristics attributed to the Schottky barrier between the HfO_2_ and the TiN electrode. The asymmetric characteristics in the forward and reverse directions are attributed to the asymmetric structures of the top and bottom electrodes. No hysteresis in the current–voltage characteristics was observed as a function of the bias sweep direction, whereas the capacitance–voltage characteristics exhibited typical butterfly hysteresis characteristics, as reported in [5]. No difference was observed in the current–voltage characteristics as a function of the polarization direction, which implies the same noise behavior. To determine the transport mechanism, ln (I/V) versus V^0.5^ and ln (I/V^2^) versus 1/V are plotted in Figure 4a and 4b, respectively [9]. ln (I/V) versus V^0.5^ follows a straight line from 0.7 to 3.2 V in Figure 4a, which implies that the leakage current phenomenon is associated with the Poole–Frenkel (PF) mechanism caused by the oxide traps and vacancies in the HfO_2_ FE layer (see Figure 1b) [7,8,9]. In contrast, the Fowler–Nordheim (FN) mechanism caused by tunneling through the HfO_2_ layer becomes dominant at voltages higher than 2.5 V, as shown in Figure 4b [11].

**Figure 5:**
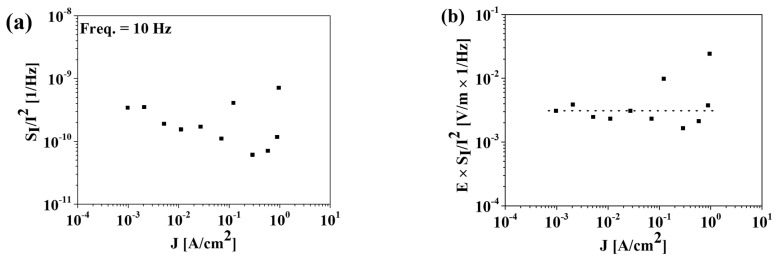
(**a**) *S_I_*/*I*^2^ versus *J* and (**b**) *E* × *S_I_*/*I*^2^ versus *J* at *f* = 10 Hz.

Figure 3b shows the bias-dependent LFN characteristics of the MFM device measured in the frequency (*f*) range of 4–103 Hz. The bias voltage was applied from 0.5 to 3 V to the top TiN electrode and for the bottom TiN electrode. The normalized current noise power spectral density (*S_I_*/*I*^2^) curve exhibited a 1/*f* noise shape, as shown in Figure 3b, regardless of the bias voltage, which is consistent with the noise results of the reported MIM capacitors and MFIS FET with positive polarization [6,7,8,9]. The noise levels (*S_I_*/*I*^2^) decreased while the bias voltage and measurement frequency increased. The *S_I_*/*I*^2^ values were inversely proportional to the current density, as shown in Figure 5a. Figure 5b shows *E* × *S_I_*/*I*^2^ versus the current density, from which no dependence on the current density is observed. Therefore, we suggest that the origin of the noise is primarily related to the PF emission mechanism, following the model given in [9,12]:

(1) $$E\times \frac{S_{I}}{I^{2}}\propto \beta^{2}=constant\;value,$$

where *β* is the field enhancement factor. The *β* value increases as thermal field emission is added to the PF emission [9,12]. The *β* value of the HfO_2_ MFM capacitor fabricated in this study was approximately three orders of magnitude lower than the one reported in [9]. In addition, no increase in the *β* value as a function of the current density was observed, indicating that the thermal emission was negligible and that only a PF mechanism was responsible for the leakage behavior.

**Figure 6:**
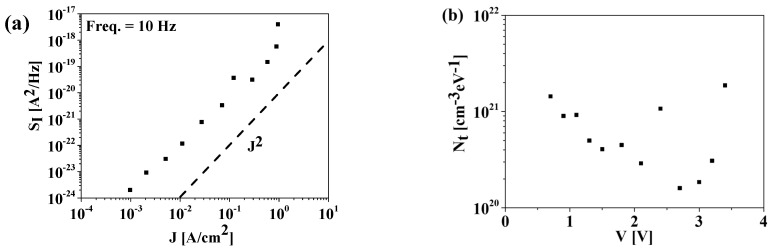
(**a**) Current power spectral density (*S_I_*) as a function of current density (*J*) at *f* = 10 Hz for the fabricated MFM capacitor. The dashed line indicates *J^2^*. (**b**) Oxide trap density (*N_t_*) versus voltage extracted from *S_I_* measurements.

The *S_I_* versus the current density is plotted in Figure 6a to investigate the FE material quality. The *S_I_* is proportional to *J^2^* in the current density range below *J* = 10^0^ A/cm^2^, which can be expressed by [6,13,14]:

(2) $$S_{I}=\frac{BJ^{2}}{f}$$

where *B* is the trap-related value, which depends on film quality. The estimated *B* values of the fabricated devices range from 8 × 10^17^ to 3 × 10^18^. These values are comparable to those reported for SrTiO_3_ MIM capacitors [6]. 

**Figure 7:**
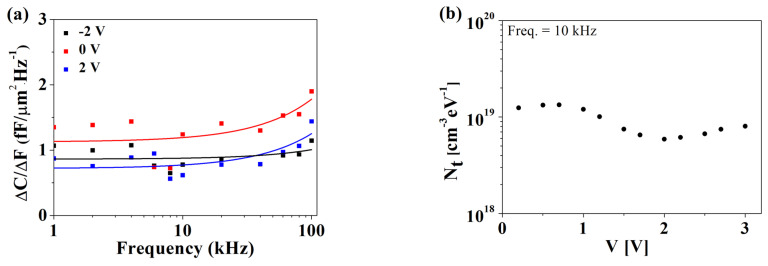
(**a**) Δ(C– F) versus measurement frequency and (**b**) oxide trap density (*N_t_*) calculated using the Δ (C– F slope) method and measured at 10 kHz.

The oxide trap density (*N_t_*) can be extracted from the measured *S_I_* using the following equations [15,16]:

(3) $$N_{t}=\frac{A\alpha fS_{I}}{a^{2}I^{2}kT}\;$$

where *A* is the capacitor area (=7.85 × 10^−5^ cm^2^), α is the oxide tunneling attenuation given by $\frac{4\pi }{h}\sqrt{2qm^{*}\phi_{B}}$, a is the blocking area (= *πr^2^*, where *r* is half of the oxide thickness [15,16]), *kT* is the thermal energy, *h* is Planck’s constant, *q* is the electron charge, m* is the effective mass, and *Φ_B_* is the barrier height. With m* = 0.15 × *m*_0_ (*m*_0_ is the electron mass) [17] and *Φ_B_* = 1.8 eV [9], and α is calculated as 6.3 × 10^7^ cm^−1^. Consequently, *N_t_* versus the voltage is plotted in Figure 6b. The derived *N_t_* was as low as 1.6 × 10^20^ cm^−3^·eV^−1^, comparable to that reported in a previous study [15]. For comparison, the *N_t_* characteristics were also derived from the difference in the slope of the capacitance–frequency (C–F) curves, that is, the Δ(C–F slope) method [18]. Figure 7a,b shows the Δ(C–F) characteristics and the extracted *N_t_* versus voltage. Notably, the Δ(C–F slope) method results in relatively lower *N_t_* values compared to other methods [18]. Therefore, the difference observed between the two methods in this study is not atypical.

## 4. Conclusions

An HfO_2_-based MFM capacitor was fabricated and characterized using I–V and noise measurements. The device exhibited a constant of *E* × *S_I_*/*I*^2^ noise values and noise shapes of 1/*f*, indicating that the dominant transport mechanism is a trap-related PF emission caused by trapping in the oxide defects. The oxide trap density, *N_t_*, extracted from the noise measurements was as low as 1.6 × 10^20^ cm^−3^·eV^−1^. The important finding is that the transport mechanism of the MFM capacitor itself has no dependency on the polarization direction, whereas the asymmetric MFIS configuration has different mechanisms depending on the bias voltage polarity. The MFM capacitor exhibited similar behavior to that of the positive polarization case of the MFIS device.

## Data Availability

Data supporting the findings of this study are available from the corresponding author upon reasonable request.
